# *Staphylococcus aureus* ventilator-associated pneumonia in patients with COVID-19: clinical features and potential inference with lung dysbiosis

**DOI:** 10.1186/s13054-021-03623-4

**Published:** 2021-06-07

**Authors:** Gennaro De Pascale, Flavio De Maio, Simone Carelli, Giulia De Angelis, Margherita Cacaci, Luca Montini, Giuseppe Bello, Salvatore Lucio Cutuli, Gabriele Pintaudi, Eloisa Sofia Tanzarella, Rikardo Xhemalaj, Domenico Luca Grieco, Mario Tumbarello, Maurizio Sanguinetti, Brunella Posteraro, Massimo Antonelli

**Affiliations:** 1 Dipartimento Di Scienze Biotecnologiche Di Base, Cliniche Intensivologiche E Perioperatorie, Università Cattolica del Sacro Cuore, Rome, Italy; 2 Dipartimento Di Scienze Dell’Emergenza, Anestesiologiche e della Rianimazione, Fondazione Policlinico Universitario A. Gemelli IRCCS - Università Cattolica del Sacro Cuore Largo A. Gemelli 8, 00168 Rome, Italy; 3 Dipartimento Di Scienze Di Laboratorio E Infettivologiche, Fondazione Policlinico Universitario A. Gemelli IRCCS, Rome, Italy; 4 Dipartimento Di Sicurezza E Bioetica, Università Cattolica del Sacro Cuore, Rome, Italy; 5 Dipartimento Di Biotecnologie Mediche, Università Di Siena, Siena, Italy; 6 Dipartimento Di Scienze Mediche E Chirurgiche, Fondazione Policlinico Universitario A. Gemelli IRCCS, Rome, Italy

**Keywords:** COVID-19, Ventilator-associated pneumonia, Lung microbiota, *Staphylococcus aureus*, Bronchoalveolar lavage

## Abstract

**Background:**

Hospitalized patients with COVID-19 admitted to the intensive care unit (ICU) and requiring mechanical ventilation are at risk of ventilator-associated bacterial infections secondary to SARS-CoV-2 infection. Our study aimed to investigate clinical features of *Staphylococcus aureus* ventilator-associated pneumonia (SA-VAP) and, if bronchoalveolar lavage samples were available, lung bacterial community features in ICU patients with or without COVID-19.

**Methods:**

We prospectively included hospitalized patients with COVID-19 across two medical ICUs of the Fondazione Policlinico Universitario A. Gemelli IRCCS (Rome, Italy), who developed SA-VAP between 20 March 2020 and 30 October 2020 (thereafter referred to as cases). After 1:2 matching based on the simplified acute physiology score II (SAPS II) and the sequential organ failure assessment (SOFA) score, cases were compared with SA-VAP patients without COVID-19 (controls). Clinical, microbiological, and lung microbiota data were analyzed.

**Results:**

We studied two groups of patients (40 COVID-19 and 80 non-COVID-19). COVID-19 patients had a higher rate of late-onset (87.5% versus 63.8%; *p* = 0.01), methicillin-resistant (65.0% vs 27.5%; *p* < 0.01) or bacteremic (47.5% vs 6.3%; *p* < 0.01) infections compared with non-COVID-19 patients. No statistically significant differences between the patient groups were observed in ICU mortality (*p* = 0.12), clinical cure (*p* = 0.20) and microbiological eradication (*p* = 0.31). On multivariable logistic regression analysis, SAPS II and initial inappropriate antimicrobial therapy were independently associated with ICU mortality. Then, lung microbiota characterization in 10 COVID-19 and 16 non-COVID-19 patients revealed that the overall microbial community composition was significantly different between the patient groups (unweighted UniFrac distance, *R*^2^ 0.15349; *p* < 0.01). Species diversity was lower in COVID-19 than in non COVID-19 patients (94.4 ± 44.9 vs 152.5 ± 41.8; *p* < 0.01). Interestingly, we found that *S. aureus* (log_2_ fold change, 29.5), *Streptococcus anginosus* subspecies *anginosus* (log_2_ fold change, 24.9), and *Olsenella* (log_2_ fold change, 25.7) were significantly enriched in the COVID-19 group compared to the non–COVID-19 group of SA-VAP patients.

**Conclusions:**

In our study population, COVID-19 seemed to significantly affect microbiological and clinical features of SA-VAP as well as to be associated with a peculiar lung microbiota composition.

**Supplementary Information:**

The online version contains supplementary material available at 10.1186/s13054-021-03623-4.

## Introduction

Since its first detection in China, the severe acute respiratory syndrome coronavirus 2 (SARS-CoV-2)-caused pneumonia, known as coronavirus disease 2019 (COVID-19), has become an unprecedented global pandemic [1]. Although many SARS-CoV-2 infected individuals undergo a mild disease [2], a significant proportion of hospitalized patients require admission to the intensive care unit (ICU) [3]. In this setting [4], mechanical ventilation (MV) is commonly used to provide supportive care [5], especially in patients with acute respiratory distress syndrome (ARDS), which is a well-established feature of COVID-19 pathophysiology [6]. While up to 50% of ventilated patients do not survive SARS-CoV-2 infection [7], ICU patients have a greater risk of secondary infection or superinfection by bacterial pathogens compared to patients in mixed ward/ICU settings [8]. It is plausible that SARS-CoV-2 infection impairs pulmonary immune responses against bacteria [9] or alters the dynamics of inter-microbial interactions [10], thereby leading to enhanced growth of pathogenic species. It is also plausible that co-infection exacerbates the lung damage triggered by SARS-CoV-2 and, then, facilitate pathogen’s systemic dissemination [7]. To date it is unclear whether specific co-infecting pathogens are associated with poor outcomes [11], as well as they correlate with the microbial community in the lung of patients suffering from SARS-CoV-2 infection [12]. A recent study investigating the lung-tissue microbiota characteristics of 20 deceased patients with COVID-19 (80% were mechanically ventilated) found a bacterial community enriched with *Acinetobacter* species [13], which include carbapenem-resistant *A. baumannii* [14]. Concomitantly, Kreitmann et al. reported that *Staphylococcus aureus* (SA) accounted for ~ 70% of bacteria isolated from early sampled lower respiratory tract of COVID-19 patients, who required mechanical ventilation for ARDS [15]. No significant difference in day-28 mortality was observed according to the presence of co-infection, and this finding was consistent with the receipt of appropriate antibiotic treatment (all SA isolates were methicillin susceptible) in co-infected patients [15]. Similarly, in critically ill influenza patients superinfected by bacterial pathogens, SA was the most common pathogen [16], whereas much less virulence to SA isolates might be required for bacterial superinfection in cases of preceding influenza infection [17]. Thus, any colonizing SA isolate may induce bacterial superinfection, suggesting that an altered balance between commensals and potential pathogens in a virus-conditioned lung microbiota may contribute to aggravate SARS-CoV-2-induced pneumonia.

The aim of this study was to investigate clinical features of SA ventilator-associated pneumonia (SA-VAP) in ICU patients with or without COVID-19. We also investigated the lung microbiota of patients for whom lower respiratory tract samples were available to identify bacterial community features related to COVID-19.

## Methods

### Study setting and design

This observational study prospectively included hospitalized patients with COVID-19 across two medical ICUs of the Fondazione Policlinico Universitario A. Gemelli IRCCS (Rome, Italy), who developed SA-VAP between 20 March 2020 and 30 October 2020 (thereafter referred to as cases). A control group of SA-VAP patients admitted to the same study sites between the calendar years 2017 and 2019 was also included and used as a historical non–COVID-19 comparator group (in this case prospectively collected data were retrospectively analyzed). For both groups, electronic patient records and microbiology laboratory data were used to identify patients and to retrieve clinical data (e.g., presence of one or more comorbidities), microbiological results (i.e., from cultures of bronchoalveolar lavage [BAL] fluid or other relevant samples), receipt and/or type of antimicrobial treatment(s), and outcomes. Following identification, patients from COVID-19 group were 1:2 matched with those from non–COVID-19 group (control patients), based on the Simplified Acute Physiology Score II (SAPS II) [18] (within 5 points at ICU admission), and the Sequential Organ Failure Assessment (SOFA) score [19] (within 2 points at SA-VAP diagnosis). In cases of multiple possible controls, choice fell on those patients who shared closest SAPS II and SOFA score values. During case–control matching, investigators were blinded to cases’ outcomes.

The study was performed in accordance with the Declaration of Helsinki and was approved by the Ethics Committee of the Fondazione Policlinico A. Gemelli IRCCS (reference number 23703/19). A written informed consent or proxy consent was obtained according to committee recommendations.

### Definitions and outcomes

Acute kidney injury, ARDS, septic shock, or VAP were defined according to previously reported criteria [20–23]. The SA-VAP was defined as bacteremic when the diagnosis of SA-VAP coincided with *S. aureus* isolation in at least one blood culture in the absence of other specified source of bacteremia [24]. The primary outcome was the mortality in the ICU, whereas secondary outcomes were the clinical cure, microbiological cure, and in-hospital mortality. Clinical cure of SA-VAP was defined as the complete resolution of all signs and symptoms of infection by the end of targeted therapy (i.e., appropriate antimicrobial therapy), including no progression of any previous chest-radiography abnormalities. Microbiological cure was defined as the lack of SA growth in cultures from subsequently collected respiratory tract and/or blood samples. Empirical antimicrobial therapy, called inappropriate initial antimicrobial treatment (IIAT), consisted into administering an antimicrobial agent against which the patient’s SA isolate was not susceptible (see above). When judgements were discordant (about 2% of patients), the reviewers reassessed the data and reached a consensus decision (see Additional file 1: Figure S1 e-diagram).

### Microbiology laboratory testing

#### Microbiota characterization

We characterized the microbiota of BAL fluid samples collected from a subset of SA-VAP patients in both COVID-19 and non–COVID-19 groups. All samples were stored at − 80 °C until processing, which only for samples from COVID-19 patients was performed in a biosafety level 3 cabinet. Total DNA was extracted in a strictly controlled separate and aseptic laboratory workplace. Briefly, 5 ml of each sample was centrifuged, and the resulting pellet was carefully resuspended in sterile phosphate-buffered saline. This suspension was used to extract DNA with the DANAGENE MICROBIOME Saliva DNA kit (DanaGen-BioTed S.L., Barcelona, Spain) according to manufacturer’s instructions. The extracted DNA from each sample was checked for quality by agarose gel electrophoresis and for concentration by the Qubit™ 4.0 fluorometer (Thermo Fisher Scientific, Rodano, Italy) measurement using the Qubit dsDNA HS (High Sensitivity) Assay kit (Life Technologies, Monza, Italy). For each sample, the V3‒V4 hypervariable regions of the 16S rRNA gene was amplified using forward and reverse primers that contained the sequences 5′-TCGTCGGCAGCGTCAGATGTGTATAAGAGACAGCCTACGGGNGGCWGCAG3′ and 5′-TCTCGTGGGCTCGGAGATGTGTATAAGAGACAGGACTACHVGGGTATCTAATCC-3′, respectively [25]. The resulting amplicons were purified using Agencourt AMPure XP beads (Beckman Coulter, Milan, Italy) and then barcoded using the Nextera XT Index kit (Illumina, San Diego, CA, USA). Indexed amplicons were diluted to reach relative equimolar concentrations with one another, and the resulting library was sequenced on a MiSeq® instrument (Illumina) using a 2 × 300 paired-end configuration according to manufacturer’s recommendations. To increase the base-diversity degree, we added an internal control (PhiX v3; Illumina) to the library as previously described [26]. Sequencing reads have been submitted to the NCBI Sequence Read Archive (PRJNA693784). After demultiplexing of raw sequencing reads, FastQ sequences were analyzed according to the QIIME 2 (Quantitative Insights into Microbial Ecology 2) bioinformatics pipeline [27]. Briefly, FastQ sequences were trimmed to remove primers and barcodes, and were then quality filtered (4202769 in total; 1470182 and 2640530 reads from COVID-19 and non–COVID-19 patient samples, respectively). We processed the sterile saline used for sample collection as an extraction and sequencing control, which yielded median 151901 reads per sample. Using the DADA2 algorithm [28], removal of chimeras led to obtain amplicon sequence variants (ASVs), which underwent a taxonomic annotation via a pre-fitted scikit-learn classifier based on SILVA 132 reference database [https://www.scikit-learn.org/stable/]. Finally, we removed sequences from mitochondrial DNA or sequences from microbial taxa with less than 0.01% representability [29].

### Statistical analyses

Clinical data analysis was performed using MedCalc Statistical Software version 16.4.3 (MedCalc, Ostend, Belgium), whereas data were graphed using GraphPad Prism version 6.0 (GraphPad Software, San Diego, CA). Continuous data were presented as median (interquartile range [IQR]), whereas categorical data were presented as counts and proportions. Differences between groups for continuous data were assessed using either Student’s *t*-test (normally distributed) or Mann–Whitney *U*-test (non-normally distributed), whereas those for categorical data were assessed using the chi-square test or Fisher’s exact test as appropriate. Odds ratios and 95% confidence intervals were calculated. Variables with a *P* value < 0.1 in univariable analysis were included in multivariable analyses, which were conducted using stepwise logistic regression. Microbial community data analysis was performed in R studio version 4.0.2 (https://www.rstudio.com/) using the phyloseq package [30]. For each sample, alpha diversity was determined by calculating diversity indices (number of observed species, Shannon, the Simpson’s inverse, and the Pielou’s species evenness) at a rarefaction depth of 105,851 sequences, and significant differences between groups were assessed using the Wilcoxon nonparametric test. To assess compositional (dis)similarity between samples, beta diversity was determined by calculating the unweighted UniFrac distance and was visualized as a principal coordinates analysis (PCoA) plot [31], and significant differences between groups were assessed using PERMANOVA. Relative abundances of microbial community members between groups were also calculated, whereas the analysis of differentially abundant taxa was conducted using the DESeq2 package [32]. In all analyses, a *P* value < 0.05 was set as the statistical significance threshold.

## Results

### Characteristics of patients who developed SA-VAP in the ICU

**Fig. 1:**
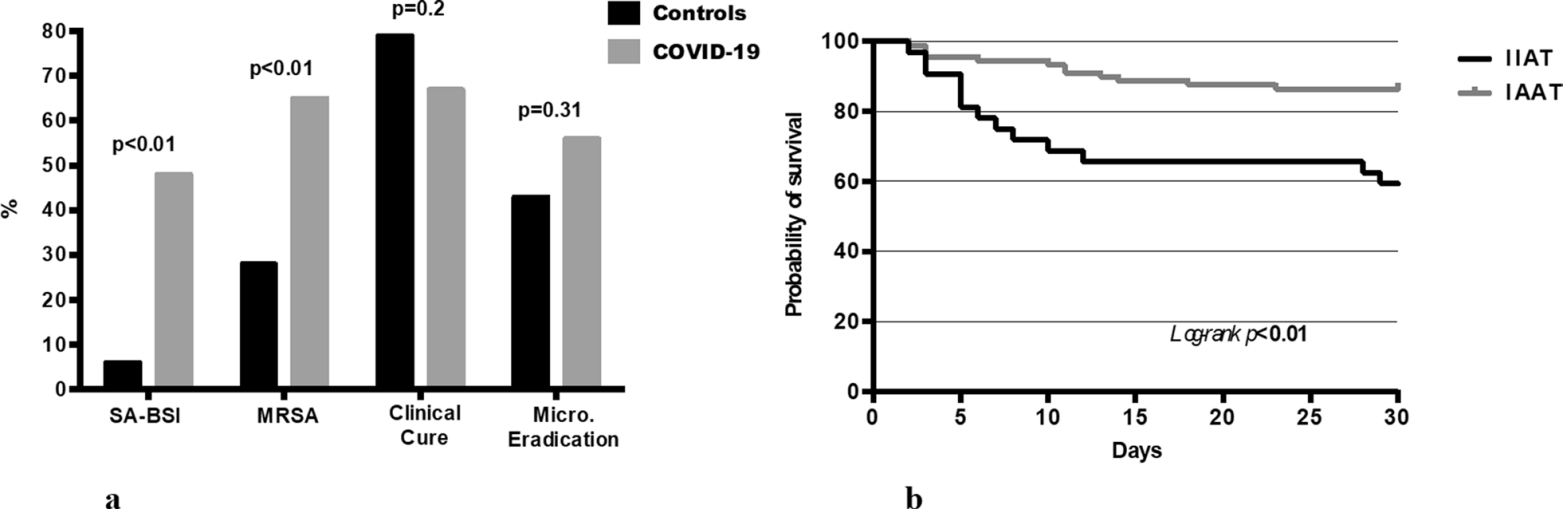
**a** Microbiological and clinical features of SA VAP in COVID-19 patients and controls. **b** Kaplan–Meier curve showing the impact of IIAT (black line) on ICU mortality. SA: Staphylococcus aureus; BSI: bloodstream infection; MRSA: methicillin-resistant Staphylococcus aureus; Micro: microbiological; VAP: ventilator-associated pneumonia; IIAT: initial inadequate antibiotic therapy; IAAT: initial adequate antibiotic therapy

During the study period, 40 (43.5%) of 92 VAPs were due to SA and thus selected for the analysis (Additional file 1: Figure S1 e-diagram). COVID-19 patients with SA-VAP (*n* = 40) were compared with non–COVID-19 patients with SA-VAP (*n* = 80), who were used as controls (Table 1). At the time of SA-VAP diagnosis, we did not observe significant differences between patient groups in terms of demographics or main comorbidities. Furthermore, rate of septic shock, acute kidney injury requiring renal replacement therapy, duration of ICU stay, and use of mechanical ventilation before SA-VAP were similar in both groups. Regarding SA-VAP features, COVID-19 patients were more likely to have a late-onset infection (35/40 [87.5%] vs. 51/80[63.8%]; *P* = 0.01), methicillin-resistant infection (26/40 [65%] vs. 22/80 [27.5%]; *P* < 0.01), or bacteremic infection (19/40 [47.5%] vs. 5/80 [6.3%]; *P* < 0.01) than non–COVID-19 patients (see also Fig. 1a) (see also Additional file 2: e-Table S1). Regarding antistaphylococcal antimicrobial agents, linezolid was most frequently used to treat COVID-19 patients (24/40 [60%] vs. 8/80 [10%];* P* < 0.01), whereas other (non-oxacillin or non-vancomycin) agents to treat non–COVID-19 patients (2/40 [5%] vs. 34/80 [42.5%]; *P* < 0.01). Interestingly, no statistically significant differences between COVID-19 and non–COVID-19 groups were found regarding the receipt of IIAT or the duration of antimicrobial treatment (Table 1).

**Table 1:** Characteristics of 120 study patients diagnosed with SA-VAP

	Total (*n* = 120)	COVID-19 (*n* = 40)	Non–COVID-19 (*n* = 80)	*P* value
*Demographics*				
Age, years	63 [52–70]	64 [58–70]	62 [47–74]	0.21
Male, *N* (%)	92 (76.7)	33 (82.5)	59 (73.8)	0.36
SAPS II score	38 [30–43]	38 [32.5–46.5]	38 [29–40.5]	0.15
*Comorbidities*				
Cardiovascular disease, *N* (%)	20 (16.7)	7 (17.5)	13 (16.3)	0.8
Diabetes, *N* (%)	19 (15.8)	8 (20.0)	11 (13.8)	0.43
COPD, *N* (%)	19 (15.8)	7 (17.5)	12 (15.0)	0.79
Chronic renal failure, *N* (%)	11 (9.2)	3 (7.5)	8 (10.0)	0.75
Immunosuppression, *N* (%)	8 (6.7)	4 (10.0)	4 (5.0)	0.44
Neoplasm, *N* (%)	7 (5.8)	4 (10.0)	3 (3.8)	0.22
*Characteristics at diagnosis*				
Respiratory failure, *N* (%)^a^	112 (93.3)	40 (100)	72 (90%)	0.1
Length of stay in ICU, days	8 [7–12]	11.5 [4–19]	8 [7–9]	0.29
Duration of mechanical ventilation, days	6 [5–10]	9 [4–17]	6 [5–7]	0.14
SOFA score^b^	7 [5–9]	7 [4–9]	7 [5–9]	0.51
Previous antibiotics, *N* (%)^c^	67 (55.8)	25 (62.5)	42 (52.5)	0.39
*Characteristics after diagnosis*				
Length of stay in ICU, days	12 [6.5–23.5]	11 [6–22]	14 [6.5–24]	0.46
Duration of mechanical ventilation, days	7 [3–11]	7 [2.5–11]	6.5 [3–11]	0.86
*SA-VAP features*				
Late-onset infection, *N* (%)	86 (71.7)	35 (87.5)	51 (63.8)	**0.01**
Methicillin-resistant infection, *N* (%)	48 (40.0)	26 (65.0)	22 (27.5)	** < 0.01**
Bacteraemic infection, *N* (%)	24 (20.0)	19 (47.5)	5 (6.3)	** < 0.01**
*Complications*^b^				
Septic shock, *N* (%)	65 (54.2)	22 (55.0)	43 (53.8)	1
Acute kidney injury requiring CRRT, *N* (%)	12 (10.0)	4 (10.0)	8 (10.0)	1
*Antistaphylococcal antimicrobial therapy*				
Oxacillin, *N* (%)	31 (25.8)	9 (22.5)	22 (27.5)	0.66
Vancomycin, *N* (%)	21 (17.5)	5 (12.5)	16 (20.0)	0.45
Linezolid, *N* (%)	32 (26.7)	24 (60.0)	8 (10.0)	** < 0.01**
Other antimicrobials, *N* (%)^d^	36 (30.0)	2 (5.0)	34 (42.5)	** < 0.01**
Initial inadequate antimicrobial therapy, *N* (%)	32 (26.7)	13 (32.5)	19 (23.8)	0.38
Duration of antimicrobial therapy, days	8 [6–10]	9.5 [7–10]	7 [5–10.5]	0.12
Vancomycin MIC < 1 mcg/mL, *N* (%)	61 (50.8)	22 (55.0)	39 (48.8)	1
Linezolid MIC < 2 mcg/mL, *N* (%)	29 (24.2)	11 (27.5)	18 (22.5)	0.51
*Outcomes*				
Clinical cure, N (%)	90 (75.0)	27 (67.5)	63 (78.9)	0.2
Microbiological cure, N (%)^e^	42 (50.6)	16 (43.2)	26 (56.5)	0.31
ICU death, *N* (%)	30 (25.0)	14 (35.0)	16 (20.0)	0.12
In-hospital death, *N* (%)	32 (26.7)	14 (35.0)	18 (22.5)	0.19

Data are presented as median [IQR], unless otherwise indicated

Significant values are in bold (*P* values < 0.1)

*SA* Staphylococcus aureus, *VAP* ventilator-associated pneumonia, *SAPS II* Simplified Acute Physiology Score, *COPD* chronic obstructive pulmonary disease, *ICU* Intensive Care Unit, *SOFA* Sequential Organ Failure Assessment, *CRRT* continuous renal replacement therapy, *MIC* minimal inhibitory concentration; *IQR* interquartile range

^a^72 out of 80 controls were admitted to ICU with respiratory failure due to different origins (community-acquired/nosocomial pneumonia, chest trauma, ARDS due to septic shock, COPD exacerbations, coma with suspicion of inhalation). Remaining 8 patients were admitted due post-surgical haemorragic shock (*n* = 3) and major trauma (*n* = 5)

^b^The day of VAP diagnosis

^c^Antibiotics received ≥ 48 h during previous 30 days: *amoxicillin/clavulanic acid, piperacillina/tazobactam, ceftolozane/tazobactam, ceftriaxone, cefepime, meropenem, amikacin, vancomycin, linezolid, colistin, tigecycline, azytromicin*, *levofloxacin*

^d^Amoxicillin clavulanic-acid (*n* = 19), quinolones (*n* = 15), trimethoprim sulphametoxazole (*n* = 2)

^e^Microbiological eradication was evaluated in 83 pts (37 COVID-19+ and 46 controls)

### Outcomes and predictors of ICU mortality

Despite being higher among COVID-19 patients, ICU mortality did not significantly differ between COVID-19 or non–COVID-19 groups (35% vs. 20%, *P* = 0.12) (Table 1). Similarly, no differences between groups were observed regarding clinical cure and microbiological eradication of SA-VAP (see also Fig. 1a). Comparing alive (*n* = 90) or deceased (*n* = 30) patients on univariate analysis (Table 2) showed that age (*P* = 0.01), SAPS II (*P* < 0.01), and the SOFA score (*P* = 0.06) were associated with higher risk of death. Similarly, neoplasm (*P* = 0.06), COVID-19 (*P* = 0.08), bacteremic infection (*P* = 0.01), methicillin-resistant infection (*P* = 0.01), and IIAT (*P* ≤ 0.01) were more likely to occur in patients who died. Furthermore, deceased patients were more likely to receive linezolid treatment (*P* = 0.02) than alive patients were, whereas oxacillin treatment was associated with lower risk of death (*P* = 0.01). However, on multivariable logistic regression analysis (Table 2), SAPS II (adjusted odds ratio [OR], 1.08; 95% confidence interval [CI], 1.03–1.14; *P* < 0.01) and IIAT (adjusted OR, 4.63, 95% CI, 1.56–13.7; *P* < 0.01) were found to be independently associated with ICU mortality. The multivariable Cox-regression model confirmed that IIAT significantly increased the risk of ICU mortality (hazard ratio [HR], 3.5; 95% CI, 1.62–7.72), which was consistent with the Kaplan–Meier survival curve analysis results (*P* < 0.01) shown in Fig. 1b.

**Table 2:** Univariate and multivariable analysis of factors associated with ICU mortality

	Patients with SA-VAP		Univariate analysis		Multivariable analysis	
	Alive (*n* = 90)	Deceased (*n* = 30)	*P* value	OR (95% CI)	*P* value	OR (95% CI)
*Demographics*						
Age, years	60 [50–68]	69 [60–71]	0.01	1.04 (1.01–1.08)	0.11	1.04 (0.99–1.09)
Male sex, *N* (%)	69 (76.7)	23 (76.7)	1	1 (0.38–2.66)	–	–
SAPS II score	36 [29–39]	41.5 [36–53]	< 0.01	1.07 (1.03–1.11)	** < 0.01**	**1.08 (1.03–1.14)**
*Comorbidities*						
Cardiovascular diseases, *N* (%)	12 (13.3)	8 (26.7)	0.11	2.33 (0.85–6.42)	–	–
Diabetes, *N* (%)	16 (17.8)	3 (10.0)	0.29	0.51 (0.14–1.9)	–	–
COPD, *N* (%)	14 (15.6)	5 (16.7)	0.89	1.37 (0.49–3.79)	–	–
Chronic renal failure, *N* (%)	7 (7.8)	4 (13.3)	0.38	1.82 (0.49–6.73)	–	–
Immunosuppression, *N* (%)	5 (5.6)	3 (10.0)	0.42	1.89 (0.42–8.43)	–	–
Neoplasm, *N* (%)	3 (3.3)	4 (13.3)	0.06	4.46 (0.94–21.2)	0.1	4.99 (0.76–33)
*Presenting features and therapy*						
COVID 19+ , *N* (%)	14 (15.6)	26 (86.7)	0.08	2.15 (0.92–5.04)	0.83	0.86 (0.22–3.44)
ICU LOS before VAP, days	8 [7–11]	7 [6–14]	0.21	1.01 (0.99–1.04)	–	–
MV duration before VAP, days	6 [6–10]	5.5 [4–13]	0.24	1.01 (0.99–1.04)	–	–
SOFA score^a^	7 [4–8]	8 [5–10]	0.06	1.15 (0.99–1.33)	0.99	1 (0.81–1.23)
Septic shock, *N* (%)^a^	46 (51.1)	19 (63.3)	0.24	1.65 (0.71–3.86)	–	–
AKI requiring CRRT, *N* (%)^a^	9 (10.0)	3 (10.0)	1	1 (0.25–3.96)	–	–
Bacteraemic infection, *N* (%)	11 (12.2)	13 (43.3)	0.01	3.43 (1.33–8.84)	0.31	2.1 (0.51–8.36)
Late VAP, *N* (%)	63 (70.0)	23 (76.7)	0.48	1.41 (0.54–3.67)	–	–
Methicillin-resistant infection, *N* (%)	30 (33.3)	18 (60.0)	0.01	3 (1.28–7.04)	0.77	0.82 (0.21–3.16)
Vancomycin MIC < 1 mcg/mL, N (%)	47 (52.2)	14 (46.7)	0.43	1.42 (0.86–2.34)	–	–
Linezolid MIC < 2 mcg/mL, *N* (%)	20 (22.2)	9 (30.0)	0.27	1.61 (0.69–3.75)	–	–
Vancomycin treatment, *N* (%)	17 (18.9)	4 (13.3)	0.48	0.66 (0.2–2.15)	–	–
Linezolid treatment, *N* (%)	19 (21.1)	13 (43.3)	0.02	2.86 (1.18–6.9)	0.47	1.68 (0.41–6.86)
Oxacillin treatment, *N* (%)	28 (31.1)	3 (10.0)	0.01	0.25 (0.07–0.88)	0.06	0.14 (0.02–1.06)
Other treatments, *N* (%)^b^	25 (27.8)	11 (36.7)	0.5	1.35 (0.57–3.22)	–	–
IIAT, *N* (%)	16 (17.8)	16 (53.3)	< 0.01	8.29 (2.15–12.9)	** < 0.01**	**4.63 (1.56–13.7)**

Data are presented as median [IQR], unless otherwise indicated

Significant values are in bold (*P* values < 0.1)

*SA* Staphylococcus aureus, *VAP* ventilator-associated pneumonia, *OR* odds ratio, *CI* confidence interval, *SAPS II* Simplified Acute Physiology Score, *COPD* chronic obstructive pulmonary disease, *LOS* length of stay, *ICU* Intensive Care Unit, *MV* mechanical ventilation, *SOFA* Sequential Organ Failure Assessment, *AKI* acute kidney injury, *CRRT* continuous renal replacement therapy, *MIC* minimal inhibitory concentration, *IIAT* Initial Inadequate Antimicrobial Therapy, *IQR* interquartile range, *ROC* receiver operating characteristic, *AUC* area under the curve, *SE* standard error

We included all variables in the multivariable logistic regression if they reached *p* ≤ 0.1 on univariate analysis. A stepwise selection procedure was used to select variables for inclusion in the final model. ROC curve analysis was used to assess the goodness of the final logistic regression model (AUC ± SE = 0.85 ± 0.05 with 95%CI 0.77–0.91; chi-square statistics *p* < 0.001)

^a^The day of VAP diagnosis

^b^Amoxicillin clavulanic-acid (*n* = 19), quinolones (*n* = 15), trimethoprim sulphametoxazole (*n* = 2)

### SA-VAP related lung microbiota profiles of COVID-19 or non–COVID-19 patients

**Fig. 2:**
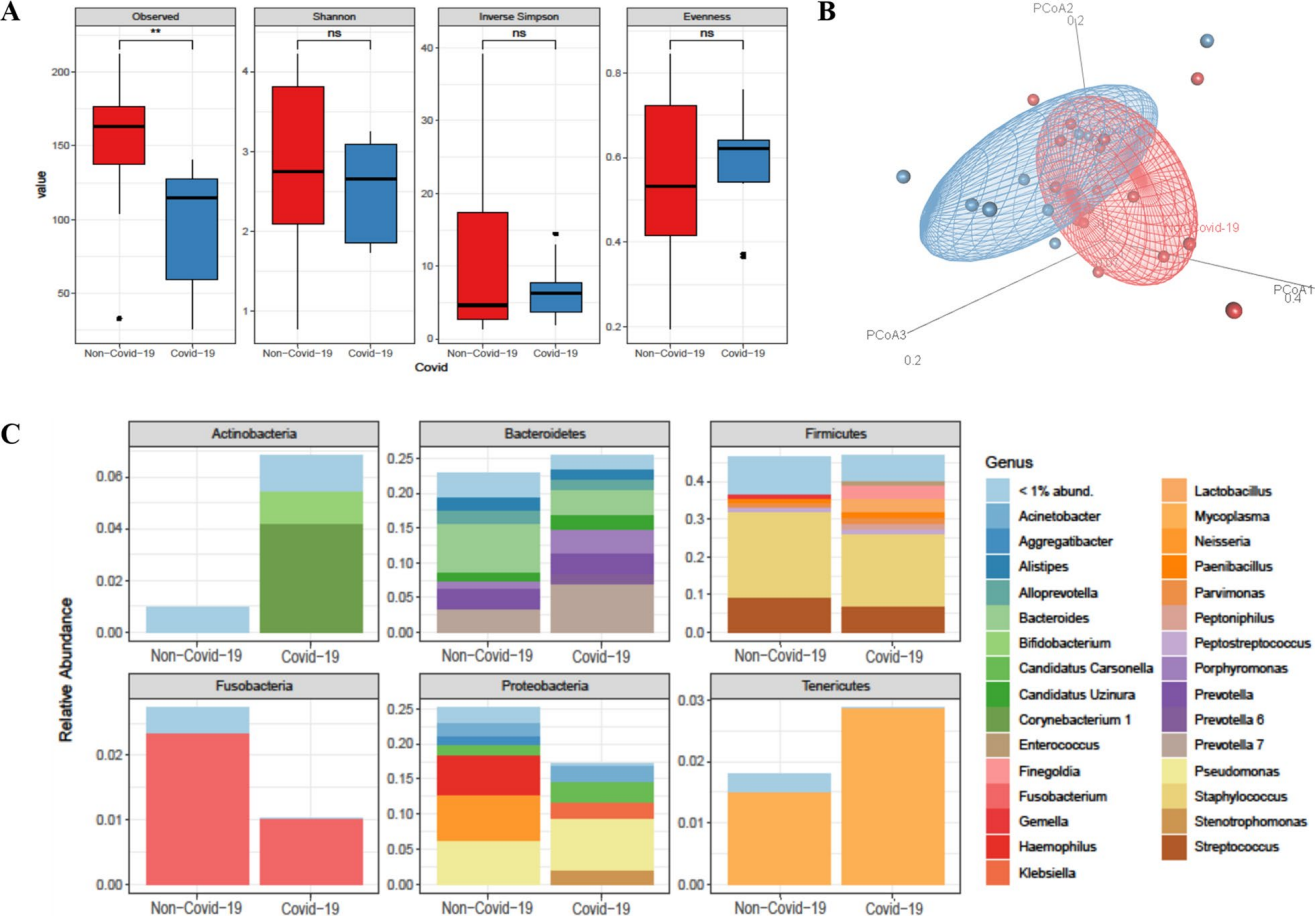
Lung microbiota composition and diversity indices in patients with S. aureus respiratory infection. Alpha diversity (**a**) and Unweighted Unifrac Beta diversity (**b**) were analysed on the basis of the dataset normalized to 105,851 reads per sample. The two groups compared were defined by SARS-CoV-2 positive infection. The differences between groups were assessed by Wilcoxon nonparametric test showing a lower alpha diversity in COVID-19 group compared to patients from the non-COVID-19 group (Observed index: 94.4 ± 44.9 vs. 152.5 ± 41.8; P = 0.001). No significant differences were observed in the Shannon (*P* = 0.421), Simpson’s inverse (*P* = 0.979), or Pielou's evenness (*P* = 0.938) indices. Permutational multivariate analysis of variance (PERMANOVA) showed significant differences between the microbial communities of the two groups (*R*^2^ 0.15349, *P* = 0.004). Panel C shows the mean of the relative abundances of the 30 more represented genera within the six major phyla that compose the lung bacterial community of COVID-19 and non-COVID-19 groups

**Fig. 3:**
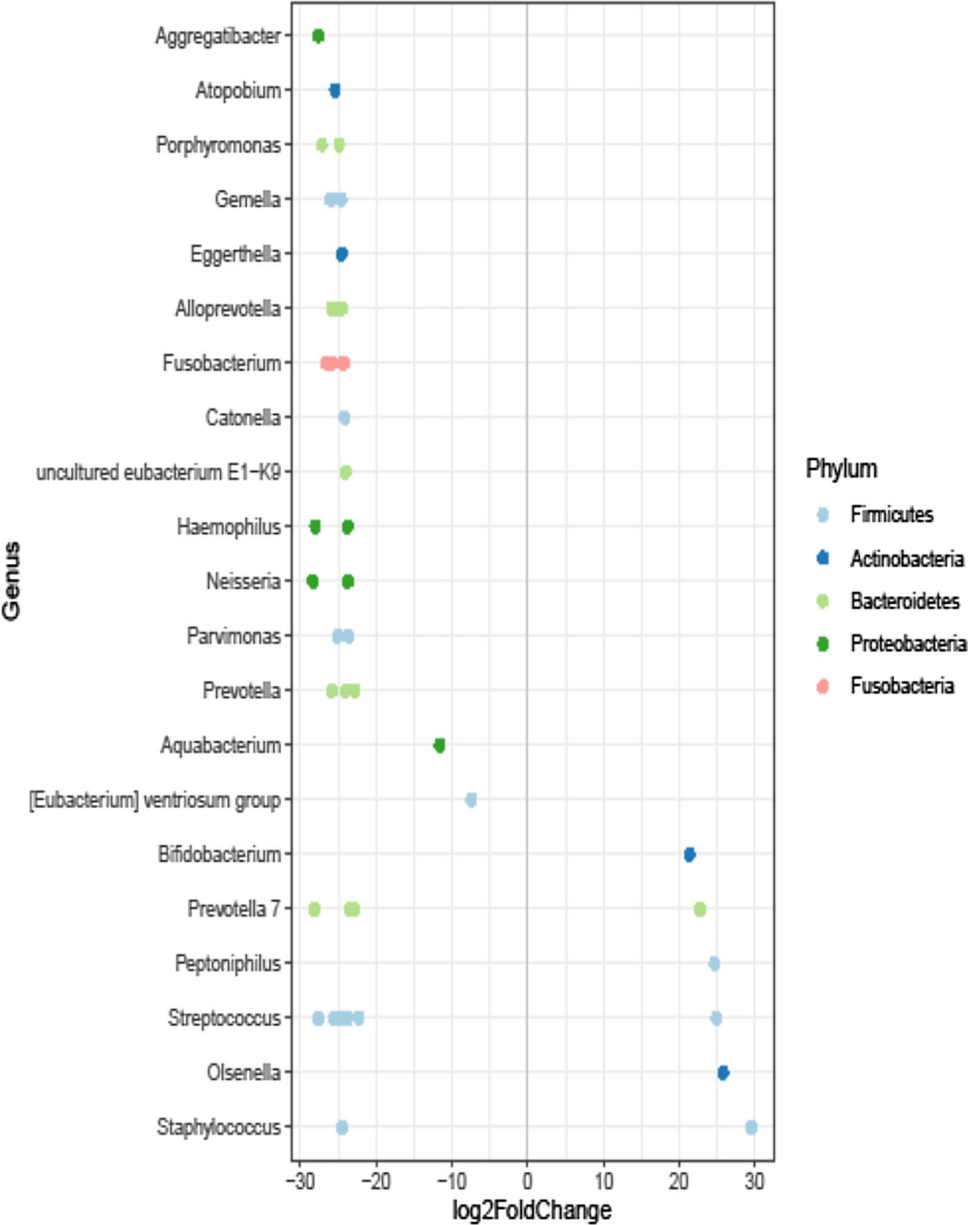
Differential abundances between patients with concomitant SARS-CoV-2 and S. aureus respiratory infection and SARS-CoV-2-negative patients with S. aureus infection. The analysis of differentially abundant taxa was assessed using the DESeq2 package. In all analyses, a *P* value < 0.05 was set as the statistical significance threshold. Positive values of log2 Fold change represent genera significantly more abundant in Covid-19 group

Our sequencing strategy of BAL samples obtained from COVID-19 (*n* = 10) or non–COVID-19 (*n* = 16) patients provided 3,928,074 sequences in total (ranging from 105,857 to 202,048 sequences per sample), which were classified in 542 ASVs representing 11 bacterial phyla. The most prominent phyla were (in alphabetic order) Actinobacteria, Bacteroidetes, Firmicutes, Fusobacteria, Proteobacteria, and Tenericutes, together accounting for 99.9% of all sequences. Although previous antimicrobial prescriptions (90% vs. 100%, *p* = 0.81) and mechanical ventilation days (9 days [6–19] in COVID pts vs 7.5 days [4–14.5] in non-COVID patients; *p* = 0.46) were similar in the two groups, we observed that the overall composition of the lung microbiota in COVID-19 patients was significantly different from that in non–COVID-19 patients (unweighted UniFrac distance, *R*^2^ 0.15349, *P* = 0.004) (Fig. 2). However, the UniFrac-derived PCoA plot showed overlapping between the two microbial communities, indicating that samples of patient groups’ communities shared features that may account for clustering on different spatial levels. According to observed species, diversity was lower in patients from the COVID-19 group compared to patients from the non–COVID-19 group (94.4 ± 44.9 vs. 152.5 ± 41.8; *P* = 0.001) (Fig. 2). Conversely, no significant differences were observed in the Shannon (*P* = 0.421), Simpson’s inverse (*P* = 0.979), or Pielou's species evenness (*P* = 0.938) indices. We compared the taxa at phylum and genus level between COVID-19 and non–COVID-19 groups (Fig. 2). At phylum level, Tenericutes were relatively more abundant in patients with COVID-19 (2.8% vs. 1.7%), whereas Actinobacteria were present only in patients with COVID-19 (6.7%). Conversely, Fusobacteria and Proteobacteria were relatively less abundant in COVID-19 patients (1.0% vs. 2.7% and 16.8% vs. 24.9%, respectively). In both groups, relative abundances of Bacteroidetes and Firmicutes were similar (around 45.0%). At genus level, *Porphyromonas* (Bacteroidetes) and *Prevotella 7* (Bacteroidetes) were relatively more abundant in COVID-19 patients (3.5% and 7.0% vs. 1.1% and 3.4%, respectively). Conversely, *Alistipes* (Bacteroidetes), *Bacteroides* (Bacteroidetes), and *Fusobacterium* (Fusobacteria) were relatively less abundant in COVID-19 patients (2.1%, 7.1%, and 2.3% vs. 1.2%, 3.8%, and 1.0%, respectively). Interestingly, *Bifidobacterium* (Actinobacteria), *Corynebacterium 1* (*Actinobacteria*), *Prevotella 6* (*Actinobacteria*), *Enterococcus* (Firmicutes), *Lactobacillus* (Firmicutes), *Peptoniphilus* (Firmicutes), *Klebsiella* (Proteobacteria) (2.0%), and *Stenotrophomonas* (Proteobacteria) were present only in COVID-19 patients. Conversely, *Gemella* (Firmicutes), *Aggregatibacter* (Proteobacteria), *Haemophilus* (Proteobacteria), and *Neisseria* (Proteobacteria) were present only in non–COVID-19 patients. We also used DESeq analysis to identify taxa for which the relative abundance was significantly different in patients with or without COVID-19. In addition to *Peptoniphilus* (log_2_ fold change, 24.6), *Prevotella 7* (log_2_ fold change, 22.7), and *Bifidobacterium dentium* (log_2_ fold change, 21.3), we found that *Staphylococcus aureus* (log_2_ fold change, 29.5), *Streptococcus anginosus* subspecies *anginosus* (log_2_ fold change, 24.9), and *Olsenella* (log_2_ fold change, 25.7) were significantly enriched in the COVID-19 group compared to the non–COVID-19 group of SA-VAP patients (Fig. 3).

## Discussion

We studied clinical features of ICU patients with early or late SA-VAP who were categorized as having (*n* = 40) or not having (*n* = 80) COVID-19, which is a well-known predisposing condition to bacterial co-infection or superinfection [8, 33]. Patients without COVID-19 had been hospitalized in the ICU before the end of 2019, the date COVID-19 has become globally pandemic [1]. While COVID-19 or non-COVID-19 patient groups did not significantly differ in terms of ICU mortality (*P* > 0.05), we found that patients with COVID-19 significantly differed from patients without COVID-19 in terms of clinically relevant SA-VAP features such as a bacteremic (*P* < 0.01) or a methicillin-resistant (*P* < 0.01) infection. We therefore tried to relate these findings to the lung microbial community features that were investigated in subgroups of COVID-19 (*n* = 10) or non-COVID-19 (*n* = 16) patients.

A blood culture results’ evaluation study in COVID-19 patients presenting to New York city (NYC) hospitals revealed that SA was the second (after *Escherichia* coli) most common cause of bacteremia among COVID-19 patients [34]. In another recent study [35], SA has been the most detected respiratory pathogen in SARS-CoV-2 positive (7/10; 70%) compared with SARS-CoV-2 negative (18/53; 34%) patients. Again, in a bacterial infections’ analysis in 74 hospitalized patients with COVID-19 [36], 59.5% (44/74) of patients acquired superinfections, with 11 (25.0%) of 44 being VAP (caused by SA in 4/11 [36.4%]) and 16 (36.4%) of 44 being bacteremia (caused by coagulase-negative staphylococci in 7/16 [43.8%]). While 56.8% (25/44) of these superinfections occurred in ICU patients and caused worse outcomes [36], it seemed that bacteremia did not complicate SA-VAP in that study. Conversely, in a SA-bacteremia series in COVID-19 patients from two NYC hospitals, Cusumano et al. [37] identified pneumonia in 8 (19.0%) of 42 cases as a source of infection, with 5 (26.3%) of 19 patients surviving at 14 days from the first positive blood culture.

In our study, bacteremic infection was significantly more frequent in patients with than in patients without COVID-19. Several factors in COVID-19 patients may account for this finding, such as complex immune dysregulation [38], administration of immunomodulatory drugs (e.g. corticosteroids), long durations of ICU stay and MV prior to infection onset. Our data was consistent with the observed prevalence of nosocomial bloodstream infections in ICU patients [39], but it was in contrast with that reported previously [34, 40, 41]. Very recently, the OUTCOMEREA network describing clinical and epidemiological features of COVID-19 patients observed that these patients were significantly more likely to develop late-onset (> 7 days) ICU-acquired bloodstream infections compared to non-COVID-19 patients [42]. Furthermore, COVID-19 patients were found to develop more VAP than patients without COVID-19 despite sharing a similar pulmonary microbiota [43]. Until recently (i.e., in pre–COVID-19 era), bacteremic SA pneumonia was considered as relatively uncommon despite being associated with high mortality rates [39]. Among 98 pneumonia cases, 56 due to methicillin-susceptible SA (MSSA) and 42 due to methicillin-resistant SA (MRSA), studied by De la Calle et al. in 2016 [24], 7.1% (7/98) of cases were bacteremic. In another study [39] conducted in 2006 on patients from nine European countries’ ICUs, bacteremia (caused by MRSA or *A. baumannii*) accounted for 70 (14.6%) of 479 culture-documented cases of nosocomial pneumonia (465 of which were VAP). MRSA infection was an independent risk factor for the development of bacteremia, and ICU mortality was significantly higher in bacteremic (57.1%) than in non-bacteremic patients (33.0%; *P* < 0.001). Said that, it is noteworthy that, in our study, rates of bacteremic or methicillin-resistant infections were significantly higher in COVID-19 (47.5% and 65.0%, respectively) than in non-COVID-19 (6.3% and 27.5%, respectively) patients (*P* < 0.01, for both comparisons). Finally, taken together, these findings are consistent with the results from a 18 studies’ meta-analysis on 8,249 samples from COVID-19 patients [44]. The analysis showed that SA accounted for 25.6% (95% CI 15.6–39.0) of microbial pathogen/COVID-19 coinfections whereas MRSA accounted for 53.9% (95% CI, 24.5–80.9) of SA/COVID-19 coinfections.

Although the role of synergic interactions between SARS-CoV-2 and co-infecting bacteria remains unclear [11], changes in the respiratory tract microbiota (i.e., a dysbiosis status) of VAP patients [45] may reduce the resistance to colonization by potentially pathogenic bacteria [7], including antibiotic-resistant bacteria such as MRSA [46]. It is also plausible that SARS-CoV-2 infection uncovers bacterial receptors on epithelial lung cells, thereby favoring bacterial attachment, growth, and dissemination, and then increasing the risk of bloodstream infection and sepsis [7]. On the other hand, extensive antibiotic treatments in COVID-19 patients [47] may perturb gut homeostasis, allowing bacterial pathogens to cause pneumonia or other invasive infections [48]. Consistent with a significantly altered gut microbiota of COVID-19 patients compared to healthy controls [49], our 16S rRNA gene sequencing data show that the lung bacterial community of patients with COVID-19 was different from that of patients without COVID-19 (*P* = 0.004). Interestingly, *Olsenella* and *Streptococcus anginosus*, which normally inhabit the oropharynx forming biofilm to enhance bacterial adherence and thriving [50], were among the taxa significantly enriched in the COVID-19 lung microbiota together with SA. Thus, co-dominance of SA with oropharyngeal bacteria in the lung microbiota from COVID-19 patients may result from viral-induced dysbiosis and/or dysregulated immune responses [9] that, in turn, modify the interaction with other bacterial or host cells [51]. Consequently, the route by which SA-VAP progresses may differ in COVID-19 from in non-COVID-19 patients, supporting higher rates of bacteremia or other clinical features (i.e., methicillin-resistant infection) in COVID-19 patients than in non-COVID-19 patients observed by us. Otherwise, the hypothesis that hypervirulence [52] or good-fitness [17] attributes drove SA to become dominant in the lung of COVID-19 patients cannot be excluded. In our population, it was difficult to dissect changes in the lung microbiota in COVID-19 versus non–COVID-19 patients that might have occurred following the challenge with SARS-CoV-2 from changes that might have reflected differing antimicrobial susceptibility or evolving characteristics of the microbial community in the lung of patients during the study periods. However, we tried to overcome this study limitation by checking the two patient groups for potential differences in prior antimicrobial use or in duration of mechanical ventilation, which both are known to perturb the respiratory tract community and to affect the vulnerability to pneumonia in ICU patients.

In this study, ICU mortality was not statistically different between patients with and without COVID-19. Conversely, data in critically ill patients with influenza showed that the presence of coinfections, particularly SA coinfection, was an independent risk factor for mortality [53, 54]. In our patients, common profiles of clinical severity and management were found to be associated with ICU mortality, while the potential of decreased lung bacterial diversity to influence ICU mortality in COVID-19 patients was not assessed..

The present study had some limitations. First, its monocentric design could limit the applicability of our findings to non-ICU settings or to other centers. Second, the sample size was relatively small, the ratio of matched cases/controls was not 1:1 and the controls were selected during a different period (three-year period before cases). Third, the role of the immune dysregulation status (e.g. laboratory data) and of the administration of immunomodulatory drugs (e.g. corticosteroids) in the study group was not investigated, although both factors could have influenced clinical and microbiological findings. Finally, SA-VAP diagnosis was based on both tracheal aspirate and bronchoalveolar lavage samples, because the latter samples were not systematically obtained due to high risk of aerosol generation.

## Conclusions

Bacterial coinfections/superinfections represent a challenging issue in critically ill COVID-19 patients. Late-onset and methicillin-resistant SA-VAP typically occur in these patients, and are significantly associated with bloodstream dissemination. SARS-CoV-2 lung dysbiosis may explain such peculiar features, but ICU mortality remains mainly driven by the severity of clinical conditions or the prompt initiation of effective antibiotic therapy.

## Supplementary Information

**Additional file 1. Figure S1:** e-diagram.

**Additional file 2. eTable S1:** Characteristics of 48 study patients diagnosed with MRSA-VAP.

## Data Availability

The datasets used and/or analysed during the current study are available from the corresponding author on reasonable request.

## Abbreviations

COVID-19: Coronavirus disease 2019
SA: Staphylococcus aureus
VAP: Ventilator-associated pneumonia
ICU: Intensive care unit
BAL: Bronchoalveolar lavage
SAPSII: Simplified Acute Physiology Score II
SOFA: Sequential Organ Failure Assessment
SARS-CoV-2: Severe acute respiratory syndrome coronavirus 2
MV: Mechanical ventilation
ARDS: Acute respiratory distress syndrome
IIAT: Inappropriate initial antimicrobial treatment
dsDNA: *Double-stranded* Deoxyribonucleic acid
HS: High sensitivity
rRNA: Ribosomal ribonucleic acid
NCBI: National Center for Biotechnology Information
QIIME 2: Quantitative insights into microbial ecology 2
DADA: Divisive Amplicon Denoising Algorithm
ASVs: Amplicon sequence variants
IQR: Interquartile range
OR: Odds ratio
CI: Confidence interval
PCoA: Principal coordinates analysis
PERMANOVA: Permutational multivariate analysis of variance
HR: Hazard ratio
NYC: New York city
MSSA: Methicillin-susceptible *Staphylococcus aureus*
MRSA: Methicillin-resistant *Staphylococcus aureus*
