# Supplementary material for: Staphylococcus aureus ventilator-associated pneumonia in patients with COVID-19: clinical features and potential inference with lung dysbiosis
Source: Crit Care. 2021 Jun 7;25:197. doi: 10.1186/s13054-021-03623-4 (PMC8182737; doi:10.1186/s13054-021-03623-4)
Supplement: Supplementary file 1 — Additional file 1. Figure S1: e-diagram. [file 13054_2021_3623_MOESM1_ESM.docx]

***Staphylococcus aureus* ventilator-associated pneumonia in patients with COVID-19**

**Clinical features and lung microbiota analysis**

**Electronic Supplement**

*Microbiology laboratory testing*

Combined nasal and oropharyngeal swabs were obtained from COVID-19 patients to detect one or more SARS-CoV-2 specific nucleic acid targets [1] by the Korean Ministry of Food and Drug Safety approved Allplex™ 2019-nCoV assay (Arrow Diagnostics S.r.l., Genova, Italy), which is a real-time reverse-transcriptase–polymerase-chain-reaction (RT-PCR) based assay for SARS-CoV-2 RNA detection [2]. A positive RT-PCR result (i.e., a cycle threshold value of <40) was used to confirm COVID-19 diagnosis, which in turn relied on the presence of fever and/or lower respiratory tract symptoms and on lung imaging (e.g. chest radiography) features consistent with SARS-CoV-2 pneumonia [3]. To diagnose SA-VAP [4], BAL fluids or endotracheal aspirates were obtained from COVID-19 or non–COVID-19 patients at the time of clinical deterioration in acute physiology or new infiltrates on chest radiography. Samples were considered positive for bacterial isolates using a threshold of ≥10^4^ colony forming units in the fluid or ≥10^5^ in the aspirate cultured on standard media. Isolates were identified as *S. aureus* by matrix-assisted laser desorption/ionization time-of-flight mass spectrometry (BioTyper® system; Bruker Daltonics, Bremen, Germany). Antimicrobial susceptibility testing (AST) of SA isolates was performed using the microdilution broth method with the VITEK® 2 AST system (bioMérieux, Marcy l’Étoile, France), and minimum inhibitory concentration (MIC) values were interpreted according to current European Committee on Antimicrobial Susceptibility Testing breakpoints (version 10.0, http://www.eucast.org/clinical_breakpoints/). When available, BAL fluid samples were aliquoted and stored at −80°C for respiratory microbiota characterization.

**Bibliography**

1. Corman VM, Landt O, Kaiser M, Molenkamp R, Meijer A, Chu DK, et al. Detection of 2019 novel coronavirus (2019-nCoV) by real-time RT-PCR. Euro Surveill. 2020;25(3):2000045. doi: 10.2807/1560-7917.ES.2020.25.3.2000045.
2. Cheng MP, Papenburg J, Desjardins M, Kanjilal S, Quach C, Libman M, Dittrich S, Yansouni CP. Diagnostic testing for severe acute respiratory syndrome-related coronavirus 2: a narrative review. Ann Intern Med. 2020;172(11):726-734. doi: 10.7326/M20-1301.
3. Wu Z, McGoogan JM. Characteristics of and important lessons from the coronavirus disease 2019 (COVID-19) outbreak in China: summary of a report of 72 314 cases from the Chinese Center for Disease Control and Prevention. JAMA. 2020;323(13):1239-1242. doi: 10.1001/jama.2020.2648. PMID: 32091533.
4. Kalil AC, Metersky ML, Klompas M, Muscedere J, Sweeney DA, Palmer LB, et al. Management of adults with hospital-acquired and ventilator-associated pneumonia: 2016 clinical practice guidelines by the Infectious Diseases Society of America and the American Thoracic Society. Clin Infect Dis. 2016;63(5):e61-e111. doi: 10.1093/cid/ciw353.

**e-diagram 1**

Flow-chart of case-control study inclusion process

**Microbiologically confirmed VAP in COVID-19** (n=92)

**Microbiologically confirmed VAP (3-y cohort population)** (n=468)

**Excluded:** (n=118)

♦Not matched according to SAPS II and SOFA score

**Available SA-VAP controls** (n=198)

**Excluded** (n=52)

♦ Non SA-VAP

**Excluded:** (n=270)

♦Non SA-VAP

**Patients analysed as controls (1:2)** (n=80)

**Patients analysed as SA-VAP cases** (n=40)

**Legend**

*SA*: *Staphylococcus aureus*; *VAP*: ventilator-associated pneumonia; *SAPS II:* Simplified Acute Physiology Score; *SOFA*: Sequential Organ Failure Assessment
