# Supplementary material for: Staphylococcus aureus ventilator-associated pneumonia in patients with COVID-19: clinical features and potential inference with lung dysbiosis
Source: Crit Care. 2021 Jun 7;25:197. doi: 10.1186/s13054-021-03623-4 (PMC8182737; doi:10.1186/s13054-021-03623-4)
Supplement: Supplementary file 2 — Additional file 2. eTable S1: Characteristics of 48 study patients diagnosed with MRSA-VAP. [file 13054_2021_3623_MOESM2_ESM.docx]

**eTable 1. Characteristics of 48 study patients diagnosed with MRSA-VAP**

|  | | **No. (%) of patients** | | | |
| --- | --- | --- | --- | --- | --- |
|  | **Total**  **(*n* = 48)** | | **COVID-19**  **(*n* = 26)** | **Non–COVID-19**  **(*n* = 22)** | ***P* value** |
| ***Demographics*** |  | |  |  |  |
| Age, years | 67 [58-71] | | 66 [59-70] | 67 [53-74] | 0.73 |
| Male, N (%) | 38 (79.2) | | 23 (88.5) | 15 (68.2) | 0.15 |
| SAPS II score | 38 [30.5-43] | | 38 [32-46] | 38 [26-40] | 0.25 |
| ***Comorbidities*** |  | |  |  |  |
| Cardiovascular disease, N (%) | 11 (22.9) | | 5 (19.2) | 6 (27.3) | 0.7 |
| Diabetes, N (%) | 7 (14.6) | | 5 (19.2) | 2 (9.1) | 0.43 |
| COPD, N (%) | 8 (16.7) | | 4 (15.4) | 4 (18.2) | 0.79 |
| Chronic renal failure, N (%) | 5 (10.4) | | 2 (7.7) | 3 (13.6) | 0.65 |
| Immunosuppression, N (%) | 5 (10.4) | | 3 (11.5) | 2 (9.1) | 1 |
| Neoplasm, N (%) | 4 (8.3) | | 3 (11.5) | 1 (4.5) | 0.6 |
| ***Characteristics at diagnosis*** |  | |  |  |  |
| Length of stay in ICU, days | 10 [6.5-21.5] | | 12 [4-23] | 10 [8-14] | 0.93 |
| Duration of mechanical ventilation, days | 6 [5-10] | | 10 [4-21] | 7 [6-10] | 0.63 |
| SOFA score * | 7 [5-9] | | 7 [4-9] | 7 [6-10] | 0.26 |
| ***Characteristics after diagnosis*** |  | |  |  |  |
| Length of stay in ICU, days | 15 [7-27] | | 13 [5-28] | 16 [9-26] | 0.38 |
| Duration of mechanical ventilation, days | 8.5 [4-14] | | 6 [2-14] | 9 [6-15] | 0.18 |
| ***SA-VAP features*** |  | |  |  |  |
| Bacteraemic infection, N (%) | 16 (33.3) | | 13 (50.0) | 3 (13.6) | **0.01** |
| ***Complications **** |  | |  |  |  |
| Septic shock, N (%) | 29 (60.4) | | 14 (53.8) | 15 (68.2) | 0.4 |
| Acute kidney injury requiring CRRT, N (%) | 8 (16.7) | | 4 (15.4) | 4 (18.2) | 0.79 |
| ***Antistaphylococcal antimicrobial therapy*** |  | |  |  |  |
| Vancomycin, N (%) | 21 (43.8) | | 5 (19.2) | 16 (72.7) | 0.12 |
| Linezolid, N (%) | 14 (29.2) | | 9 (34.6) | 5 (22.7) | 1 |
| Initial inadequate antimicrobial therapy,  N (%) | 20 (41.7) | | 10 (38.5) | 10 (45.5) | 0.77 |
| Duration of antimicrobial therapy, days | 9 [6.5-11] | | 10 [7-11] | 7 [6-11] | 0.39 |
| ***Outcomes*** |  | |  |  |  |
| Clinical cure, N (%) | 20 (41.7) | | 13 (50.0) | 7 (31.8) | 1 |
| Microbiological cure, N (%) | 20 (41.7) | | 13 (50.0) | 7 (31.8) | 1 |
| ICU death, N (%) | 18 (37.5) | | 11 (42.3) | 7 (31.8) | 0.56 |
| In-hospital death, N (%) | 18 (37.5) | | 11 (42.3) | 7 (31.8) | 0.37 |

Data are presented as median [IQR], unless otherwise indicated

*MRSA:* methicillin-resistant Staphylococcus aureus; *VAP*: ventilator-associated pneumonia; *SAPS II:* Simplified Acute Physiology Score; *COPD:* chronic obstructive pulmonary disease; *ICU:* Intensive Care Unit; *SOFA*: Sequential Organ Failure Assessment; *CRRT:* continuous renal replacement therapy; *IQR:* interquartile range.

* The day of VAP diagnosis
